# Slow Growth and Increased Spontaneous Mutation Frequency in Respiratory Deficient *afo1*^-^ Yeast Suppressed by a Dominant Mutation in *ATP3*

**DOI:** 10.1534/g3.120.401537

**Published:** 2020-10-22

**Authors:** Jing Li, Mark Rinnerthaler, Johannes Hartl, Manuela Weber, Thomas Karl, Hannelore Breitenbach-Koller, Michael Mülleder, Jakob Vowinckel, Hans Marx, Michael Sauer, Diethard Mattanovich, Özge Ata, Sonakshi De, Gregor P. Greslehner, Florian Geltinger, Bill Burhans, Chris Grant, Victoria Doronina, Meryem Ralser, Maria Karolin Streubel, Christian Grabner, Stefanie Jarolim, Claudia Moßhammer, Campbell W. Gourlay, Jiri Hasek, Paul J. Cullen, Gianni Liti, Markus Ralser, Michael Breitenbach

**Affiliations:** * State Key Laboratory of Oncology in South China, Collaborative Innovation Center for Cancer Medicine, Sun Yat-sen University Cancer Center, Guangzhou, China; † Universite Cote d’Azur, CNRS, Inserm, IRCAN, Nice, France; ‡ Department of Biosciences, University of Salzburg, Austria; § Department of Biochemistry and Cambridge Systems Biology Centre, University of Cambridge, 80 Tennis Court Rd, Cambridge CB2 1GA, UK; ** Department of Biochemistry, Charité University Medicine, Berlin Germany; †† The Molecular Biology of Metabolism Laboratory, The Francis Crick Institute, 1Midland Rd, London NW1 1AT, UK; ‡‡ Biognosys AG, Wagistrasse 21, 8952 Schlieren, Switzerland; §§ Institute of Microbiology and Microbial Biotechnology, Department of Biotechnology, University of Natural Resources and Life Sciences, Muthgasse 18, A-1190 Vienna, Austria; *** ACIB GmbH, Austrian Centre of Industrial Biotechnology, Muthgasse 11, A-1190 Vienna, Austria; ††† Department of Molecular and Cellular Biology, Roswell Park Cancer Institute, Buffalo, New York; ‡‡‡ Faculty of Biology, Medicine, and Health, University of Manchester, Manchester M13 9PT, UK; §§§ Faculty of Education, Manchester Metropolitan University, UK; **** Department of Biosciences, University of Kent, Canterbury Kent CT2 7NJ, United Kingdom; †††† Institute of Microbiology of the Czech Academy of Sciences, Videnska 1083, Prague 4 142 20, Czech Republic; ‡‡‡‡ Department of Biological Sciences, University at Buffalo, NY 14260; §§§§ Institute for Research on Cancer and Ageing of Nice (IRCAN), CNRS, INSERM, Université Côte d’Azur, 06107 NICE, France

**Keywords:** *Saccharomyces cerevisiae*, *rho-zero*, growth velocity, mutation frequency, *ATP3*

## Abstract

A yeast deletion mutation in the nuclear-encoded gene, *AFO1*, which codes for a mitochondrial ribosomal protein, led to slow growth on glucose, the inability to grow on glycerol or ethanol, and loss of mitochondrial DNA and respiration. We noticed that *afo1*^-^ yeast readily obtains secondary mutations that suppress aspects of this phenotype, including its growth defect. We characterized and identified a dominant missense suppressor mutation in the *ATP3* gene. Comparing isogenic slowly growing rho-zero and rapidly growing suppressed *afo1*^-^ strains under carefully controlled fermentation conditions showed that energy charge was not significantly different between strains and was not causal for the observed growth properties. Surprisingly, in a wild-type background, the dominant suppressor allele of *ATP3* still allowed respiratory growth but increased the petite frequency. Similarly, a slow-growing respiratory deficient *afo1*^-^ strain displayed an about twofold increase in spontaneous frequency of point mutations (comparable to the rho-zero strain) while the suppressed strain showed mutation frequency comparable to the respiratory-competent WT strain. We conclude, that phenotypes that result from *afo1*^-^ are mostly explained by rapidly emerging mutations that compensate for the slow growth that typically follows respiratory deficiency.

Respiratory-deficient yeast mutants were discovered seventy years ago (Ephrussi *et al.* 1949). Subsequent research led to the discovery of cytoplasmic inheritance and mitochondrial DNA [reviewed by (Chen and Clark-Walker 2000)]. Phenotypic traits of rho-zero mutations, which lack mitochondrial DNA, include slow growth, loss of mitochondrial respiration, and loss of the respiratory complexes of the inner mitochondrial membrane. Nuclear mutations (so-called *pet* mutations) can produce a very similar phenotype and can indirectly lead to loss of the mitochondrial DNA. Originally, it was thought that the observed slow growth of the mutants, which presented with a small colony phenotype (hence the name *petite colonie)* was caused by the presumed lack of ATP, which in those cells has to be produced exclusively by fermentative metabolism (Ephrussi *et al.* 1949). One aspect of the present paper is to demonstrate by controlled fermentation experiments that this belief is wrong. Instead, defects in other essential metabolic pathways of the mitochondria are in fact responsible for the slow growth phenotype.

Extragenic suppressor mutations of the slow growth phenotype were first described by the group of Clark-Walker (Chen and Clark-Walker 1999, 1995, 2000) who also showed that similar mutations enabled growth of *K. lactis* in the *petite* state. The mutations were located in the nuclear encoded ATPase subunits encoded by *ATP3*, *ATP2* and *ATP1*.

Spontaneous mutation frequency in respiratory-deficient yeast strains and in replicatively aged old mother cells was analyzed previously (Flury *et al.* 1976; Karthikeyan and Resnick 2005; Lang and Murray 2008), including in several recent papers (Stirling *et al.* 2014; Veatch *et al.* 2009; Dirick *et al.* 2014). All of these measurements resulted in some increase in spontaneous mutation frequency in respiratory-deficient cells compared to wild type cells, however they were not unbiased (unselected) and were not correlated with suppressors of the slow growth of the *petite* phenoytpe.

In our previous paper (Heeren *et al.* 2009) we showed that deletion of *AFO1*, a yeast gene coding for a protein of the large subunit of the mitochondrial ribosome, caused respiratory deficiency, but, however, allowed rapid growth. By comparison, a rho-zero mutant created in the same strain background, had considerable growth defects. The *afo1*^-^ mutant strain showed an increase in the replicative lifespan. This was observed using strains of the EUROSCARF yeast deletion collection.

Here, we deleted the *AFO1* gene in a haploid prototrophic yeast strain, and we genetically analyzed in crosses the influence of the *afo1*^-^ mutation and rapidly acquired suppressor mutations on the phenotype of the mutant strains. The main purpose of this communication is to present a dominant suppressor mutation of the slow growth phenotype of the respiratory deficient *afo1*^-^ mutant. Moreover, we describe additional phenotypes caused by the suppressor mutation in haploid prototrophic yeast cells. We show that the primary mutation that caused respiratory deficiency, *afo1*^-^, leads to a twofold increase in nuclear point mutation frequency, which is again reduced to near wild-type frequencies in the suppressed strain. The dominant suppressor allele is shown to be located in *ATP3*, a nuclear-encoded component of the mitochondrial F_1_ ATPase. This mutation did not increase the activity of the F_1_ ATPase. Among others, one key mitochondrial metabolic pathways needed for rapid growth is the synthesis of iron sulfur clusters (Lill *et al.* 2014; Veatch *et al.* 2009; Wu and Brosh 2012). The suppressor mutation did not increase cellular ATP production or energy charge, thus pointing to the fact that ATP and energy charge are not limiting for growth in the respiratory-deficient yeast cells.

## Materials And Methods

### Strains

All strains used in this study are summarized in Table 1.

**Table 1:** Yeast stains used in this study

Strain	Mating type	Markers	Source
**C+**	*MATalpha*	no auxotrophic markers	Brambilla 1999*^a^*
**C+ *rho-zero***	*MATalpha*	no mtDNA	this paper
**C+ *afo1*^-^**	*MATalpha*	*afo1*::*Nourseo^R^ ATP3^G348T^*	this paper *^b^*
**C+ *MATa***	*MATa*	no auxotrophic markers	this paper
**JS760**	*MATa/alpha*	*afo1*::*Nourseo^R^*/*AFO1^+^ ATP3^G348T^*/*ATP3^+^*	this paper
**JS760-6A**	*MATa*	*AFO1^+^ ATP3^G348T^*	this paper
**JS760-6B**	*MATa*	*afo1*::*Nourseo^R^ ATP3^+^*	this paper
**JS760-6C**	*MATalpha*	*AFO1^+^ ATP3^+^*	this paper
**JS760-6D**	*MATalpha*	*afo1*::*Nourseo^R^ ATP3^G348T^*	this paper
**JS765**	*MATa/MATalpha*	a cross of 6Bx6D	this paper
**C+ *ura3*^-^**	*MATalpha*	*ura3^-^*	a gift from D.Porro
**JS760-6B *his3*^-^**		same markers as JS760-6B, but *his3*::*kanMX4*	this paper

a strain GRFc of Brambilla *et al.* 1999 was renamed C+ for the purpose of the present paper.

b The mutation *ATP3^G348T^* in this strain ocurred spontaneously and was discovered during the course of this work.

### Strain constructions

C+ rho zero was made by treatment of C+ with ethidium bromide (Slonimski *et al.* 1968) and the absence of mtDNA was shown by staining with DAPI and fluorescence microscopy as described in Williamson and Fennell (Williamson and Fennell 1975).

C+ *afo1*^-^ was constructed by integrative transformation of C+ with a linear fragment of DNA encoding the *SAT1* gene conferring resistance to nourseothricin (Nourseo^R^). In particular, we used PCR primers (see list of primers) containing flanking sequences corresponding to the chromosomal copy of *AFO1* and sequences corresponding to the *Candida albicans ACT1* promoter and terminator, respectively, the ORF of *SAT1* was amplified from plasmid pSDS4 (Lettner *et al.* 2010). The *Candida albicans* sequences were used in this procedure because their promoter and terminator elements do function in *S. cerevisiae* but do not recombine with the chromosomal *S. cerevisiae* sequences. Nourseothricin resistance (Nourseo^R^) is conferred by the *SAT1* gene. We obtained a PCR product of 1344 bp. Integrative transformation into strain C+ and selection of colonies resistant to nourseothricin yielded strain C+ *afo1*^-^. Analytical PCR with primers SP cognate and ASP SAT1 showed the presence of a band of 663bp providing proof for the correct chromosomal deletion of *AFO1* in strain C+.

C+*MATa* was constructed in the following way: Strain C+ *ura3*^-^ (Branduardi *et al.* 2007) was transformed with a *URA3* selectable plasmid carrying the functional part of the yeast homothallism gene, *HO*. The resultant diploid yeast strain was now cured of the *URA3* plasmid on fluoro-orotic acid (FOA) (Sikorski and Boeke 1991; Boeke *et al.* 1987) and sporulated and complete tetrads were obtained. A spore clone that was *MATa **ura3*^-^ was mated with C+, the resulting diploid was sporulated and a spore clone was isolated by micromanipulation that was *MATa **URA3**^+^*.

JS760 resulted from mating the haploid strain just described with C+ *afo1*::*Nourseo^R^*. The four haploid strains JS760-6A, B, C, D were isolated by micromanipulation of an ascus from JS760. This tetrad is a tetratype with respect to *afo1*::*Nourseo^R^* and *ATP3**^G348T^*. Six out of ten complete tetrads obtained were tetratype as expected for two unlinked markers.

JS765: this diploid strain was obtained by a cross of JS760-6B x JS760-6D. JS760-6B *his3^-^:*In a procedure similar to the one described above for C+ *afo1*::*Nourseo^R^*, we deleted the gene, *HIS3*, in strain JS760-6B, which was necessary for testing the cloned suppressor allele *ATP3**^G348T^*. Using primers delHIS3fwd and delHIS3rev, a deletion cassette containing *kanMX4* was isolated by PCR from plasmid p416GPD *kanMX4*. The resulting DNA fragment was inserted by integrative transformation into strain JS760-6B and transformants were selected on YPD+G418 medium. The correct insertion was confirmed by analytical PCR and by re-testing transformants on SD plates revealing single colonies that were clearly *his3*^-^ auxotrophs.

### Plasmids

#### pCaAct1-Sat1 (Lettner *et al.* 2010):

This plasmid contains the *SAT1* gene coding for nourseothricin resistance and was used for the PCR construction of the deletion cassette used to disrupt *AFO1*.

#### p416GPDKanMx4:

The KanMx4 ORF was amplified from the plasmid pAH3 (Bogengruber *et al.* 2003) using the primers kanMX fwd and rev.The resulting linear DNA fragment was cloned into the vector p416GPD (Mumberg *et al.* 1995) by using EcoRI and BamHI.

*pRS313* (addgene vector database) was used to clone the *ATP3* alleles from strains C+ and C+*afo1*^-^ using the primers ATP3 fwd and ATP3rev. Basic features of this derivative of pBluescript are AmpR, *HIS3**^+^*, *CEN6** ARS4* and lacZ_a.

*pRS313ATP3+* contained the WT *ATP3**+* yeast gene under its cognate promoter cloned BamHI/XhoI as described below.

*pRS313ATP3^G348T^* contained the *ATP3**^G348T^* suppressor allel under its cognate promoter cloned BamHI/XhoI from strain JS760-6D as described below.

### Primers

All primers used in this study are collected in Table 2.

**Table 2:** Primers used in this study

Primers	Sequence
**ATP3 fwd**	AAC TCG AGT CAT CCC AAA GAG GAA GCA CCA GTA ATA AT
**ATP3 reverse**	GGA TCC TCT CTA AAA GCC GTG TCG CAG
**∆HIS3 fwd**	CTT CGA ATA TAC TAA AAA ATG AGC AGG CAA GAT AAA CGA AGG CAA AGA GTT TAT CAT TAT CAA TAC TCG
**∆HIS3 rev**	TAT ACA CAT GTA TAT ATA TCG TAT GTG CAG CTT TAA ATA ATC GGT GTC ATT AGA AAA ACT CAT CGA GCA
**Nourseo fwd**	AAC CAT TTA TAC AGA ATA GGA AAA CCA ACT AGT GCA TTA AAC TAA ACT AAA CTA AGG ATC CAG CGT CAA AAC TAG AGA
**Nourseo rev**	TAC ACA TAG GGT TTA CTA TTC TAA ACT ATA GTT ATC TTC TCT CTT ATT CTC TGC AGA GGT AAA CCC AG
**kanMX fwd**	GGA ATT CTT AGA AAA ACT CAT CGA GCA
**kanMX rev**	CGG GAT CCAT GGG TAA GGA AAA GACT

### Yeast genetics, gene manipulation and plasmid construction

Yeast media for growth and sporulation were used as described (Treco and Lundblad 2001; Lichten 2014). Yeast strains were grown on YPD (complex) or SD (synthetic minmal) media on plates or in liquid culture. As most of the experiments were performed in prototrophic strains, diploids could not be easily selected and were identified by picking colonies that were unable to mate. Sporulation was induced on SPO media for five days. Asci were digested with a solution of 0.5 mg Zymolyase 20T (Seikagaku, Japan) in 1 mL of PBS. After 5 min. the treated asci were washed and micromanipulated on YPD plates with a Singer MSM manual micromanipulator. Complete tetrads were analyzed for genetic markers and the haploid strains belonging to five tetratype tetrads were further analyzed. One of these tetratype tetrads was used for most of the more advanced phenotypic analysis experiments. For further genetic analysis of the haploid strains in crosses, the necessary matings were performed and diploids identified by screening for non-maters, as mentioned above.

Gene manipulation of yeast was performed as described in (Gardner and Jaspersen 2014).

Plasmids pRS313-*ATP3**^+^* and pRS313-*ATP3*^*G348T*^: The respective *ATP3* alleles including the presumed native promoter region (the ∼600 bp upstream region) were PCR amplified using the primers ATP3 forward and ATP3 reverse. The mutant allele was obtained from genomic DNA from strain JS760-6D. The WT ATP3 allele was obtained from strain C+. PCR products were subcloned into a pGEM-T-Easy Vector System (Promega) and further cloned into the multiple cloning site of the vector pRS313 (Sikorski and Hieter 1989) using the restriction enzymes BamHI and XhoI. The respective mutation (*ATP3*^*G348T*^) was confirmed by Sanger sequencing.

DNA sequencing of the complete genome of strain C+ *afo1*^-^ was performed by the sequencing service of the Roswell Park Cancer Institute (Buffalo, NY, USA). Bioinformatic analysis of the primary sequencing data were performed by using the methods described below for the mutation accumulation lines.

### Characterization of growth parameters of the strains

The strains were grown in SD media and the doubling times of cell numbers were determined during log phase growth. Three biological replicates were analyzed both by cell counting and by measuring optical density. Arithmetical means and standard deviations are shown.

### Bioreactor batch cultivations

The batch cultivations were performed in a 1 L bioreactor (DASGIP Parallel Bioreactor System, Eppendporf, Germany). The medium contained 1.7g Difco YNB w/o amino acids and ammonium sulfate, 5 g ammonium sulfate, and 22 g glucose monohydrate per L. Bioreactors were inoculated from an overnight culture at an optical density of 0.3. Strains were grown at 30° at pH = 5.0 kept constant by addition of NaOH. Dissolved oxygen concentration was kept above 20% saturation by controlling stirrer speed and air flow. Inlet and outlet gases were followed with the sensor provided by the bioreactor system. Samples were taken at regular intervals throughout the experiment. Biomass production was determined by measuring optical density at 600 nm and converted to cell dry mass. Concentrations of glucose, ethanol, and glycerol were determined by HPLC as decribed in Pflügl *et al.* (Pflügl *et al.* 2012).

### Metabolite measurements

Cells of the strains C+, C+ *rho-zero*, and C+ *afo1*^-^ were grown in SC media and collected in log-phase (O.D.=7.5). The cells were quenched with 25 mL of methanol precooled on dry ice, centrifuged for two min at 2000 rpm and the pellets were stored at -80°. Glass beads and 200 microL of acetonitrile/methanol (75/25 v/v) containing 0.2% formic acid were added and incubated on ice for 20 min. Cells were broken (3 × 20 sec. Fastprep, 6.5m/s) and centrifuged for 5 min at 15000 rpm at 4°. 200 microL of the supernatant were transferred to fresh tubes. The pellets were re-supended in 200 microL of H_2_O, incubated on ice for 5 min, centrifuged at 4° and 15000 rpm for 5 min and the supernatant was transferred to the vial to reach 400 microL. After another centrifugation for 5 min at 4° and 15000 rpm 50 microL of the supernatant was taken for amino acid analysis.

The remaining 350 microL were frozen and lyophilized in a Speedvac to dryness for about two h. The samples were re-suspended in 87.5 microL of 7% acetonitrile, centrifuged at 4° for 5 min at 15000 rpm, 50 microL of the supernatant was transferred to an HPLC vial for analysis of the pentose phosphate pathway intermediates.

Metabolites were quantified by liquid-chromatography selection monitoring, using a Agilent 1290 Infinity LC system, coupled to a triple quadrupole mass spectrometer (Agilent 6470), as described previously (Mülleder *et al.* 2017).

### Location of the ATP3 mutation in the structure of ATPsynthase

The mutation *ATP3**^G348T^* was localized in the yeast F(1)F(0)-ATP synthase structure (**(****Dautant *et al.* 2010****)**; PDB ID: 2WPD) by using JSmol (http://jmol.sourceforge.net/) embedded in RCSB PDB (rcsb.org). The result shows the location in the wild type structure, not in a modeled structure of the mutant.

### Measurement of F_1_ ATPase activity

Mitochondria from yeast cells (200 ml YPD cultures grown for 24 hr) were isolated by differential centrifugation. F_1_ ATPase activity was determined spectrophotometrically by using a coupled enzyme assay based on pyruvate kinase and lactate dehydrogenase. For a detailed protocol see (Magri *et al.* 2010). The F_1_ ATPase activity was calculated with the following formula:

$$\frac{\left(\Delta Abs340nm\;without\;oligomycin-\Delta Abs340nm\;with\;oligomycin\right)*V}{\varepsilon *L*v*\left[prot\right]}$$

ε= molar extinction coefficient (6.22 nm^-1^ cm^-2^);L = light path length (cm); V = reaction volume (cm^3^);v = sample volume (cm^3^); [prot]= protein concentration (mg/cm^3^)

### Measurements of oxygen uptake

Several overnight cultures (JS760-6A, JS760-6B, JS760-6C, JS760-6D, C+ and C+ *rho-zero*) were diluted to an OD600 = 0.1 in 25 ml YPD and grown to mid exponential phase at 28°, 600 rpm shaking. Oxygen consumption was analyzed in an Oxygraph 2k (Oroboros Innsbruck, Austria). From each culture 2 mL were pipetted in an O2K chamber and the measuremant was performed as described in (Grüning *et al.* 2011) and according to the manufacturer’s instructions.

### Determination of spontaneous mutation frequencies in haploid yeast strains

#### Mutation accumulation lines:

In the mutation accumulation experiments, six strains were used (see also the list of strains used in this work given above). These were: the strains of the tetrad JS760-6A, JS760-6B, JS760-C, JS760-D, and the controls C+, and C+ *rho-zero*. The tetrad JS760-6 is tetratype with respect to *afo1*::Nourseo^R^ and *ATP3**^G348T^*. All experiments were performed on YPD agar plates. Four replicate lines for each strain were propagated independently on YPD plates. To keep the number of cell divisions between bottlenecks the same across different strains, the fast growing strains JS760-6C, JS760-D, and C+ were plated to single colonies every two days, corresponding to approximately 21 cell divisions. The slow growing strains JS760-6A, JS760-6B, and C+*rho-zero* were plated to single colonies every four days, also accounting to approximately 21 cell divisions. The reason why the respiratory-competent strain JS760-6A is a slow grower is in part caused by the presence of the *ATP3**^G348T^* allele and in part by the fact that this allele leads to enhanced generation of *rho-zero petites* during growth. Taking a freshly grown single colony from the plates is defined here as a „single cell bottleneck“. We accomplished a total of 120 bottlenecks for the fast and 60 bottlenecks for the slow growers. The total number of cell divisions in the mutation accumulation lines between the ancestral and the final lines was therefore approximately 2520 for the fast-growing strains and 1260 for the slow-growing strains. Four parallel mutation accumulation lines were maintained for each of the six strains leading to a total of 24 mutation accumulation lines for sequencing.

#### DNA sequencing of the mutation accumulation lines and sequence analysis:

Genomic DNA was extracted from the six strains at the start time point and 24 (four replicated for each strain) at the endpoint of the experiments by „Yeast Master Pure“ kit (Epicenter, USA). All samples were sequenced using Illumina HiSeq 4000 PE150 platform by BGI Europe A/S (Copenhagen, Denmark). Our approach was to estimate mutation rates that are completely unbiased by selection. It has only recently become possible to do this by sequencing very large numbers of genomes at the required reading depth. The method used was based on earlier work (Lynch *et al.* 2008; Sharp *et al.* 2018; Zhu *et al.* 2014).

We performed adapter removing and quality-based trimming by trimmomatic v.0.36 (Bolger *et al.* 2014) with options ILLUMINACLIP:adapter.fa:2:30:10 SLIDINGWINDOW:5:20 MINLEN:36. The trimmed reads were mapped to the *Saccharomyces cerevisiae* S288C reference genome (Release R64-1-1) by BWA (Burrows-Wheeler transform 0.7.16a) (Li and Durbin 2009). The resulting read alignments were subsequently processed by SAMTools v.1.7 (Li *et al.* 2009), Picard tools v.1.140, and GATK v.3.6-0 (McKenna *et al.* 2010). SNVs and small indels were called by GATK HaplotypeCaller and Freebayes, respectively (Garrison and Marth 2012). The variants called by Freebayes were filtered by the VCFfilter tool from vcflib (Options: QUAL > 30&QUAL/AO > 10&SAF > 0&RPR > 1&RPL > 1). The variants existing at the start time point were filtered. In this way, we excluded sequencing errors mainly by rigorous statistical methods based on the large sequencing depth.

We then intersected the calls by both GATK HaplotypeCaller and Freebayes. We used Ensembl Variant Effect Predictor (VEP) to annotate the mutations (McLaren *et al.* 2016). All the SNVs and small indels have been manually checked by the Integrative Genomics Viewer (IGV) (Robinson *et al.* 2011). The per-base sequencing depth and the sequencing depth for each of the sixteen yeast chromosomes was calculated by SAMTools v.1.7. The copy number of mitochondrial DNA was estimated by the sequencing depth and normalized by the sequencing depth of the nuclear genome. Statistical analysis in this work was carried out in R3.6.0.

### Determination of replicative lifespans of yeast strains by microfluidics

Measurements of cell lifespans were carried out following imaging in a flow chamber modified from the Alcatras design (Crane *et al.* 2014) having traps that show higher retention of mother cells throughout their replicative lifespan (Crane *et al.* 2019). Cultures in exponential growth, in which a high proportion of cells are either newborn or have undergone only one division were introduced as described (Crane *et al.* 2014). Standard YPD medium was infused through flow chambers at 20 microL/min. Devices were mounted on a Leica inverted microscope and brightfield images captured at 5 min intervals by a Coolsnap Myo (Photometrics) camera through a 20x magnification objective. Replicative lifespans were scored manually from a randomly selected sample of cells from each genotype.

The lifespan data were statistically analyzed using Wizard (http://www.evanmiller.org/ab-testing/survival-curves.html).

### Data availability

The sequencing data obtained for mutation frequency estimation are available under BioProject ID PRJNA632985.

## Results

### Phenotypic analysis of the afo1^-^ deletion strain

In our previous paper (Heeren *et al.* 2009) we studied the phenotypic consequences of the *afo1*^-^ deletion mutant contained in the yeast deletion mutant collection EUROSCARF in the BY4741 genetic background. To re-evaluate and extend these results, the *AFO1* gene was disrupted in the BY4741 strain using the nourseothricin resistance deletion cassette (see Materials & Methods). Similarly, the *AFO1* gene was then disrupted in a prototrophic haploid strain, C+, with a different genetic background (Brambilla *et al.* 1999) using the same method. A prototrophic strain was used to avoid any complications that might arise from the auxotrophic mutations in the original BY4741 strain background. Most of the experimental results are now reported in the prototrophic strain, C+. We will occasionally also describe experiments done in the BY4741 background. The results found in the two strain backgrounds (C+ and BY4741) were identical.

**Figure 1:**
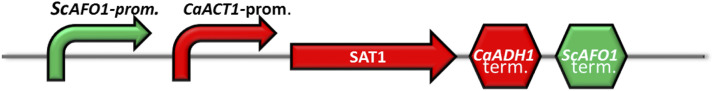
Genotype of strain C+ *afo1*^-^ after integrative transformation with Nourseo^R^ disrupting *afo1*.The figure shows the gene arrangement on chromosome VII of strain C+ after the integration of the Nourseo^R^ cassette (red symbols) in place of *AFO1*. The sequences replaced start from the start codon of the *AFO1* ORF and end at the respective stop codon. Therefore, the promoter, as well as the terminator of *AFO1*, is still intact (green symbols) and corresponds to the WT arrangement on the chromosome. The red sequences are the *Candida albicans ACT1* promoter and the *Candida albicans ADH1* terminator which flank the bacterial SAT1 gene, which confers nourseothricin resistance (Nourseo^R^).

The *AFO1* gene was replaced by the nourseothricin resistance cassette in the haploid prototrophic strain GRFc (Brambilla *et al.* 1999), renamed C+ for the present paper. The genetic manipulations needed to obtain the *afo1*^-^ deleted strain in C+ and the characterization of the correct chromosomal deletion are described in the Materials and Methods. The genetic makeup (chromosome VII) of the strain derived from this analysis is shown in Figure 1.

**Figure 2:**
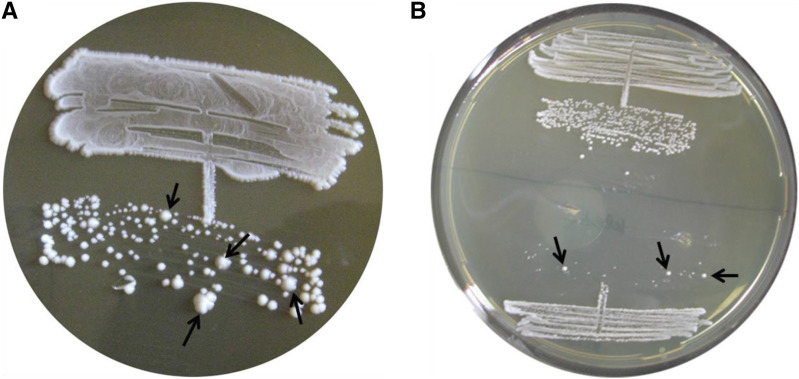
Properties of C+ *afo1* single colonies after re-streaking on YPD plates. A: Single colonies of the C+ *afo1* strain after isolation on YPD plates. All colonies are nourseothricin-resistant and unable to grow on glycerol. However, the size of the colonies (and the doubling times on glucose-based media) is very different. B: upper part: re-streaking of a large colony which produces a stable large phenotype; lower part: re-streaking of a small colony. A low percentage of the colonies was converted to large, but most of the colonies are very small. Photograph was taken after three days at 28°C. Large colonies are marked with arrows in A and B.

As expected of a respiratory-deficient mutant, the *afo1*::*Nourseo^R^* strain did not grow on glycerol. Comparison of colony size with C+ *rho-zero* and the C+ starting strain showed that the newly generated C+ *afo1*^-^ mutant strain formed a mixture of small (comparable to C+ *rho-zero*) and large colonies (comparable to WT) (Figure 2A). By comparison, the isogenic *rho-zero* strain showed only small colonies after two days growth on YPD media. Restreaking one small and one large colony of C+ *afo1*^-^ showed that the large colony phenotype was stable, while the small colony phenotype was unstable, which once again gave rise to a low percentage of large colonies (Figure 2B). This result together with examination of the colony size in the newly constructed *afo1*^-^ deletion mutant in the BY4741 background showed that the genetic instability of *afo1*^-^ mutants is independent of the strain background.

### Metabolic tests of C+ afo1^-^ and controls

**Figure 3:**
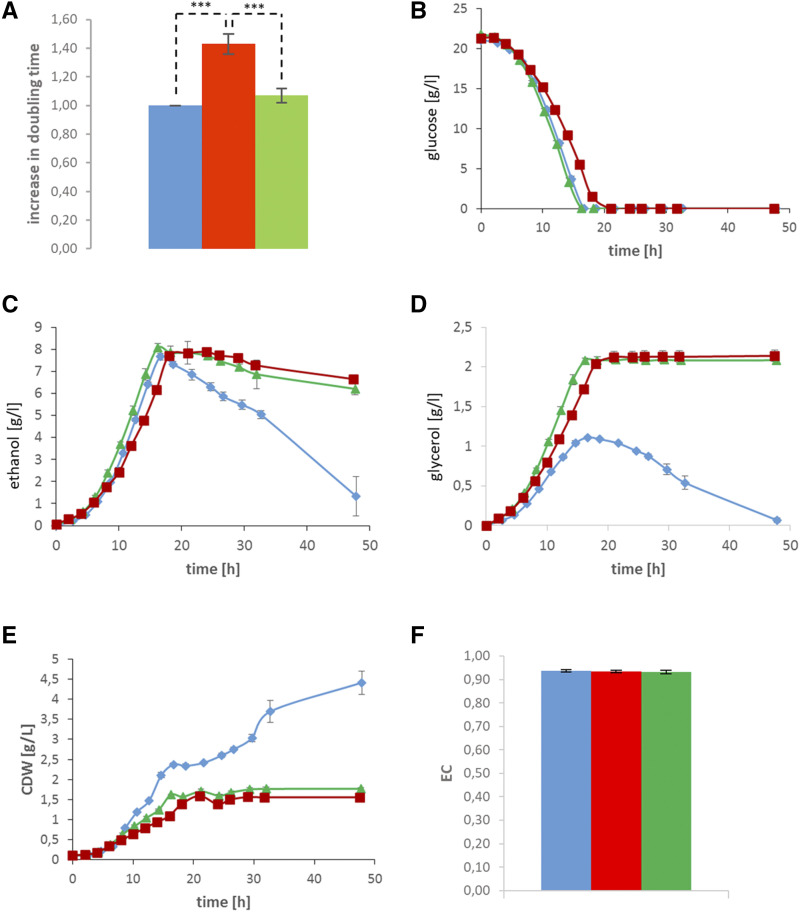
Comparison of the metabolism of C+ (blue), C+ *rho-zero* (red), and the original C+ *afo1*^-^ (green); this color code is used in 3A – 3F. A: doubling times of the three strains on synthetic complete medium with glucose as carbon source (SC medium); the doubling time of C+ *afo1* ^-^ is very similar to WT C+, the doubling time of the C+ *rho-zero* strain is significantly longer. Shown is the fold increase of doubling time relative to wild type. B: Glucose consumption of the three strains. C: Ethanol production. D: Glycerol production. The WT produces less glycerol than the non-respiring strains, and consumes it after glucose is exhausted. E: Biomass production. F: EC energy charge (a measure of ATP availability for growth and survival) is virtually identical for the three strains in midlog phase. Data are means of four independent cultures, error bars denote the standard deviation. In experiments (B-E) the results obtained with the strain C+are signifcantly different from the strains, C+ *rho-zero* and C+ *afo1*^-^ (*P* < 0.0001).

We next sought to define possible metabolic changes in the paradoxically fast growing respiratory-deficient strain C+ *afo1*^-^. The strain was batch-grown in a bioreactor fermenter (see Materials & Methods), and the relevant metabolic parameters were monitored continuously and compared with two control strains, namely the C+ respiratory competent starting strain, and the congenic *rho-zero petite* strain obtained by ethidium bromide treatment and analyzed by DAPI staining. DAPI staining also showed that the C+ *afo1*^-^ strain was free of mitochondrial DNA (data not shown). As shown in Figure 3, the metabolomic and kinetic data surveying basic metabolism were compared between the mutant C+ *afo1*^-^ fast growing strain (green) and the two controls, C+ WT (blue) and C+ *rho-zero* (red).

Figure 3A shows the generation times (doubling times) of the three strains in mid-log phase measured on SD medium. The rapidly growing isolate derived from the C+ *afo1*^-^ strain showed a similar growth rate (and was similar in many other physiological parameters) as the WT C+ strain (Figure 3A). Similar to the difference in colony size, the difference in growth rate between the rapidly growing isolate derived from the C+ *afo1*^-^ strain and the congenic *rho-zero* strain was large and statistically significant.

To further explore the metabolic properties of the suppressor, the utilization of glucose was examined by Bioreactor batch fermentation. The kinetics of glucose decline was the same in WT and in the rapidly growing isolate derived from the C+ afo^-^ strain (Figure 3B, 16 h). By comparison, the *rho-zero* strain needed about 20 h to completely ferment glucose. The rate of glucose fermentation was in agreement with the generation times shown in Figure 3A.

Ethanol production was also examined in the three strains. The maximum amount of ethanol (8 g/L, which is a typical amount for laboratory yeast strains) was reached in the WT and the rapidly growing isolate derived from the C+ *afo1*^-^ strains by 16 h growth (Figure 3C), while the congenic *rho-zero* strain reached the maximum ethanol levels by 21 h. As expected, the WT strain entered diauxie at 16 h and used up the ethanol produced within 32 h, while in the experiments performed with the non-respiring strains, the ethanol remained constant.

A different pattern of results was observed by monitoring the metabolism of glycerol. The rapidly growing isolate derived from the C^+^ *afo1*^-^ strain produced about 2.1 g/L glycerol after 16 h growth, while the *rho-zero* strain reached a similar amount at 21 h growth (Figure 3D). Both strains did not utilize glycerol as a carbon source, as expected for respiratory-deficient strains. By comparison, the WT C+ strain showed a different response with respect to glycerol, which reached a maximum of only 1.1 g/L, and which was slowly used up as a carbon source during the next 32 h.

Likewise, in terms of biomass, the WT strain reached a transient plateau of diauxie at 11 h growth and at about 15 h restarted growth (production of biomass) by using up ethanol (Figure 3E). The rapidly growing isolate derived from the C+ *afo1*^-^ strain reached maximum biomass production (1.5 g/L) at 14 h, which remained constant. The *rho-zero* strain reached the same amount of biomass sligthly later and likewise remained constant at subsequent time points.

Measuring the concentrations of the adenine nucleotides AMP, ADP, and ATP and calculating the energy charge (EC) (Andersen and von Meyenburg 1977) of midlog cells of the three strains was also performed (Figure 3F). All strains showed the expected value of EC = 0.91 with little variation. The absolute concentrations of the adenine nucleotides, in particular ATP, were very similar in the strains. Taken together, these results show that the cause for slow growth of the *rho-zero* strain during exponential phase is not due to a defect of energy charge, or adenine nucleotides. Given the rapid appearance of large colonies in the C+ *afo1*^-^ strain (and also in the corresponding strain in the BY4741 background), we tested the hypothesis that the large colonies were created due to an epigenetic switch, which is a well-known phenomenon in yeast (Liebman and Derkatch 1999). One first guess was that the rapidly growing isolates of the *afo1*^-^ deletion mutation perhaps induced epigenetic changes, but this hypothesis was dismissed because the large colony phenotype was stable (Figure 2) and did not revert to a slow-growth phenotype on media containing guanidinium hydrochloride. This drug reversibly inhibits the Hsp104 chaperone and cures most yeast prions by blocking their generation and subsequent inheritance (Chernoff *et al.* 1995; Liebman and Derkatch 1999). These experiments were performed with strains both in the C+ and in the BY4741 background. The result clearly argue against an epigenetic mechanism.

### Genomic sequencing of the strains and genetic analysis of the suppressor mutation in the rapidly growing isolates of the C+ afo1^-^ strain

To further analyze the rapid growth properties of rapidly growing isolates of the C+ *afo1*^-^ strain, we chose two different but complementary strategies: i) genomic sequencing of the strain to reveal possible secondary mutations that could cause the rapid growth phenotype (suppressor mutations), and ii) genetic analysis of the large colony (rapid growth) phenotype in crosses.

Genome sequencing of C+ *afo1*^-^ revealed a missense mutation in *ATP3*, *ATP3**^G348T^*, here also named *ATP3**^D^*, due to its dominant effect in crosses (see below). *ATP3**^G348T^* would be expected to produce a protein with the conservative amino acid change, Atp3^L116F^. We assume that the suppressor mutation occurred spontaneously during the time between disruption of the *AFO1* gene in the haploid C+ strain and first testing of the C+ *afo1*^-^ strain. As shown by Clark-Walker and his group (Chen and Clark-Walker 2000), missense mutations in the three subunits of the mitochondrial F_1_ ATPase, *ATP1*, *ATP2* and *ATP3* can suppress the partial growth defect of *rho-zero* mutations in *S. cerevisiae* and the complete growth defect in the *petite*-negative yeast, *K. lactis*. We tested this possibility by cloning and expression of the *ATP3**^G348T^* allele in a slow-growing (unsuppressed) *afo1*^-^ deletion strain, which was constructed in a cross of C+ *afo1*^-^ with the WT C+ strain. The suppressor allele restored normal growth to the C+ *afo1*^-^ strain (see below, Figure 5). The results will be discussed in a subsequent paragraph after describing the genetic analysis of C+*afo1*^-^ in a cross.

An isogenic *MATa* derivative of C+ was obtained as described in Materials and Methods.

**Figure 4:**
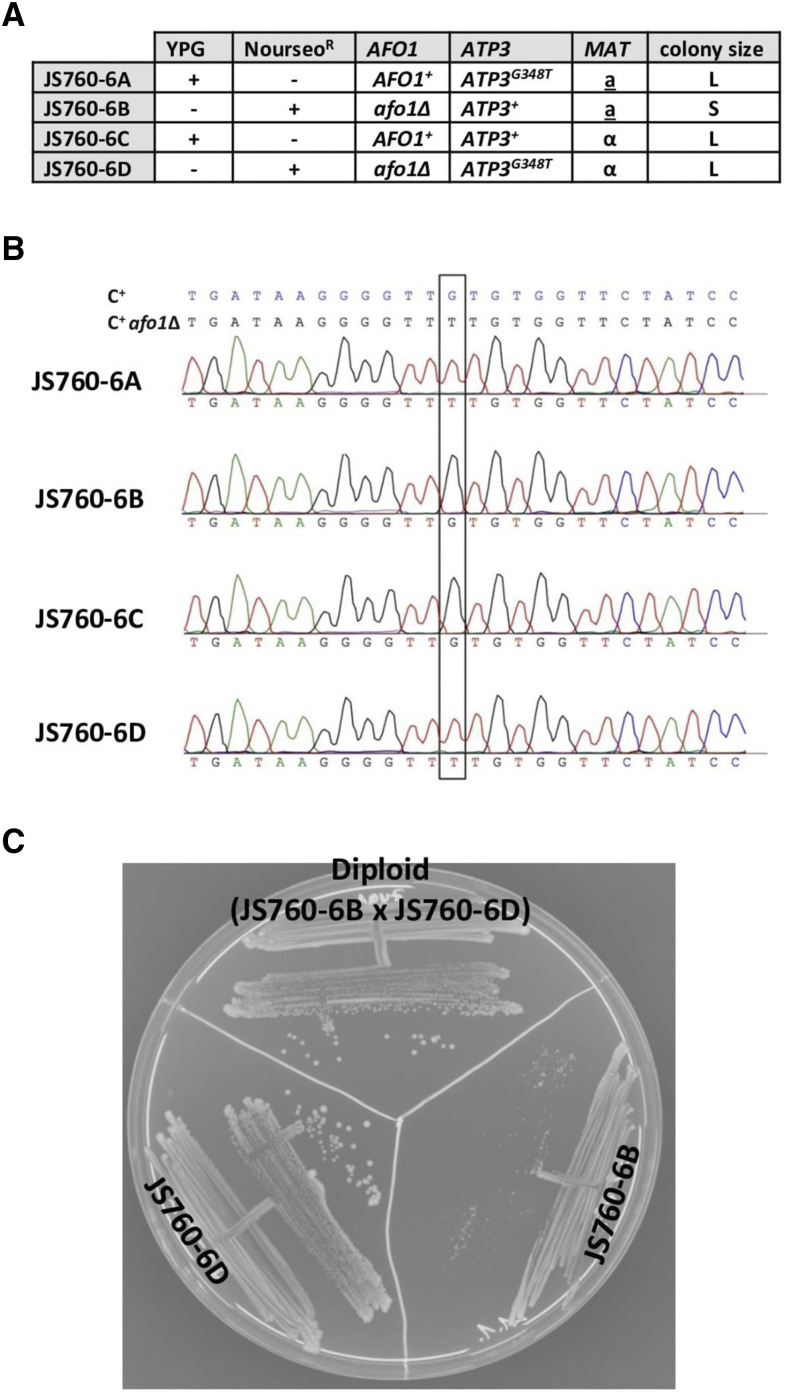
Analysis of the tetrad JS760-6. A: Properties of the four strains of the tetrad; growth on YPG, resistance to nourseothricin, sequences of the *ATP3* alleles, mating type, and colony size on YPD are monitored. B: DNA sequence of the *ATP3* genes in the strains of the tetrad. C: Dominance test for the *ATP3**^G348T^* mutation. A diploid strain (JS765 = 760-6B x 760-6D) was constructed and tested for colony size after three days on YPD.The diploid strain shows 100% large colonies. Note rare large colonies in strain JS760-6B.

Analysis of tetrads originating from the diploid strain JS760 (see Materials and Methods) showed that a second mutation was present in C+ *afo1*^-^, which caused rapid growth in *afo1* segregants forming large colonies and segregated independently of *afo1*^-^. About two thirds of the tetrads were tetratypes, as indicated by the fact that only one haploid strain in the tetrad was growing slowly (forming very small colonies), while the other members of the tetrad showed growth parameters comparable to WT. One representative tetrad (JS760-6) is shown in Figure 4A. Sequencing of the *ATP3* gene in all four member strains of this tetrad revealed that mutation *ATP3**^D^* segregated 2:2. The double mutant (JS760-6D) *afo1*^-^, *ATP3**^D^* grew rapidly, and the single mutant strain (JS760-6A) was respiratory competent (*grande*), grew rapidly, but produced a slightly elevated number of respiratory defective (*petite*) progeny on subcloning of vegetative cells. The fact that JS760-6A was respiratory competent and grew on glycerol as carbon source showed that the mutant protein Atp3^D^ apparently was functional when incorporated in the ATPsynthase structure. Figure 4B shows the *ATP3* sequences of the four strains of the tetrad. Figure 4C shows the result of a dominance test of the *ATP3**^D^* mutation in a cross of JS760-6B with JS760-6D. The picture shows 100% large colonies of the diploid strain JS765, indicating dominance of the suppressor allele *ATP3**^D^*. The picture also shows 100% large colonies of JS760-6D and a majority of small colonies with very rare large colonies after re-streaking of JS760-6B, which agrees with the original analysis of the starting strain, C+ *afo1*^-^ shown in Figure 2. In order to test the efficacy and independence of the genetic background of the cloned suppressor allele, *ATP3**^D^*, we inserted this gene in the yeast expression plasmid, pRS313 (Sikorski and Hieter 1989). As a control, we also inserted the WT *ATP3* gene in the same plasmid as described in Materials and Methods. Both alleles were expressed under the cognate *ATP3* promoter, and the selection marker for the plasmid was *HIS3*. In order to create a useful tester strain for this experiment, the unsuppressed and reasonably stable haploid strain, JS760-6B (see Figure 4C), was converted into a *his3*^-^ strain (see Materials and Methods) and transformed with the plasmids pRS313 *ATP3**^+^* and pRS313*ATP3*^*G348T*^.

**Figure 5:**
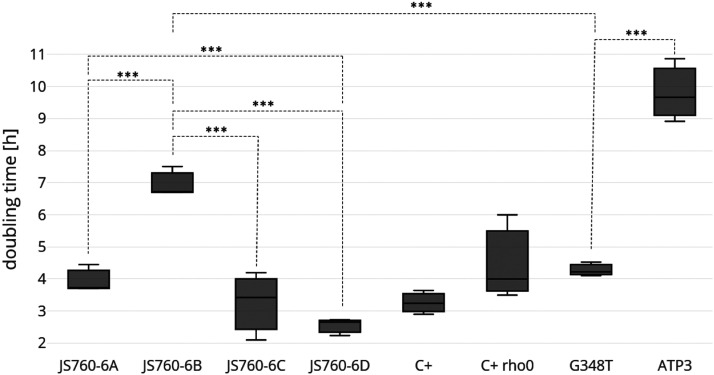
Growth rates of the four strains JS760-6A, B, C, D, and the controls C+, C+rho-zero; JS760-6B transformed with pRS313 *ATP3*^WT^, and with pRS313*ATP3**^G348T^*. All experiments were performed in liquid culture on synthetic minimal media (SD). Doubling times were determined in the exponential growth phase and the means of three independent experiments are given with standard deviations of the mean. No significant difference between WT strains and the suppressed mutant strain (760-6D) was found. However, a large and highly significant difference was observed between strains JS760-6B (unsuppressed mutant strain) and 6D (suppressed mutant strain). The C+ *rho-zero* strain is growing significantly slower than the starting WT strain C+. Strain designated *ATP3**^+^* is the JS760-6B strain expressing the WT *ATP3**^+^* gene from plasmid pRS313ATP3^+^. Strain designated G348T is the JS760-6B strain expressing the suppressor allele *ATP3**^G348T^* from plasmd pRS313ATP3^G348T^. See text for further explanations.

The results are shown in Figure 5. Large and significant differences in doubling times were found between JS760-6B and JS760-6D, which correlated well with the colony size differences shown in Figure 4C. The suppressed strain JS760-6D grew at the same rate as WT (JS760-6C) with a doubling time of 4 h, which is characteristic for the prototrophic C+ strain SD medium. Comparison of the two transformed strains, JS760-4B[*ATP3*^*G348T*^] and JS760-4B[*ATP3**^+^*] with the strains of the tetrad and the controls clearly showed that the presence of the suppressor gene, *ATP3**^G348T^*, on a plasmid could restore rapid growth to the respiratory deficient strain, JS760-4B, which the wild type gene, *ATP3**^+^*, could not. This provided proof that the major genetic factor causing rapid growth in strain JS760-6D was the *ATP3**^G348T^* allele, and was independent of the genetic background which could be somewhat different in the strains of the tetrad.

### Experiments to clarify the mechanism of suppression

**Figure 6:**
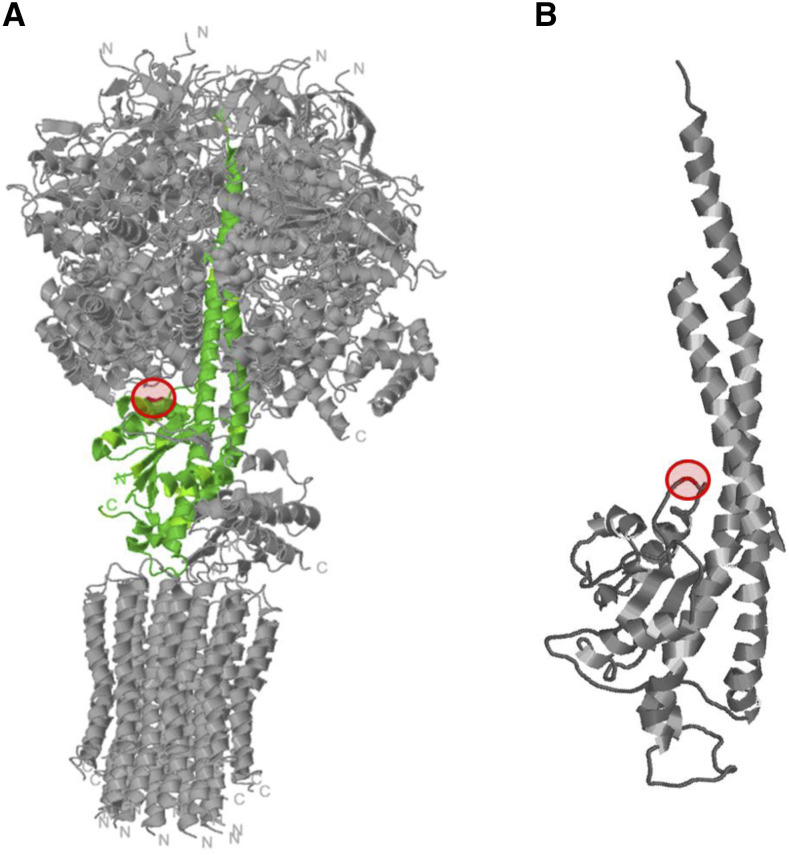
Structural model of yeast F1c_10_-ATP synthase (Dautant *et al.* 2010). A: The Atp3 subunit is shown in green with the position of the G348 (L116) residue marked in red. B: The position of the mutation is shown in an enlarged version of Atp3 structure. The mutant position lies at the interface between the rotor (Atp3) and the stator (Atp1 + Atp2).

In the next set of experiments, we aimed to test one hypothesis about the cause of rapid growth in non-respiring strains carrying *ATP3* mutant alleles that had been put forward by the group of Clark-Walker (summarized in (Chen and Clark-Walker 2000)). This hypothesis rests on the fact that all major suppressor mutations found so far share a conspicuous set of commonalities (Chen and Clark-Walker 2000): They are all located in either *ATP1*, *ATP2*, or *ATP3*; they are conservative missense mutations; they depend for activity on the intact presence of the other proteins constituting the soluble ATPase; and they are all dominant in crosses. This leads to the tentative conclusion that these mutations (even in haploids) allow the structure of the ATPase to be assembled. In our case (*ATP3*^*G348T*^), this was indeed supported by the respiratory competence of strain JS760-6A (Figure 4A). To further explore this question, we mapped the predicted amino acid change onto the structure of yeast ATP synthase ((Dautant *et al.* 2010); PDB ID 2WPD). This analysis showed that L116F lies at the interface between the Atp3 subunit („rotor“) and the Atp2 and Atp1 subunits („stator“) near the base of the Atp3 rotor (Fig.6). The location of the amino acid, L116F, is highlighted in the structural model. The other suppressor mutations found in Atp3 (Vowinckel, unpublished results) are also located at the interface between the „rotor“ and „stator“ parts of the ATPase, although they were located at the C-terminal end of the Atp3 protein stalk, near the top in the structural model. The hypothesis which was first put forward and tested by the group of Clark-Walker (Chen and Clark-Walker 2000) and posits that all of the suppresssor mutations increase the ATPase activity, and, because more ATP is hydrolysed inside the mitochondria, possibly the mitochondrial membrane potential across the inner mitochondrial membrane is increased, caused by the change in charge separation across the inner mitochondrial membrane. However, experiments later performed by the same group showed that in *K.lactis* there was no correlation with F_1_ ATPase activity, although assembly of the F_1_ ATPase complex and a minimal activity was necessary to make *K. lactis petite*-positive.

**Figure 7:**
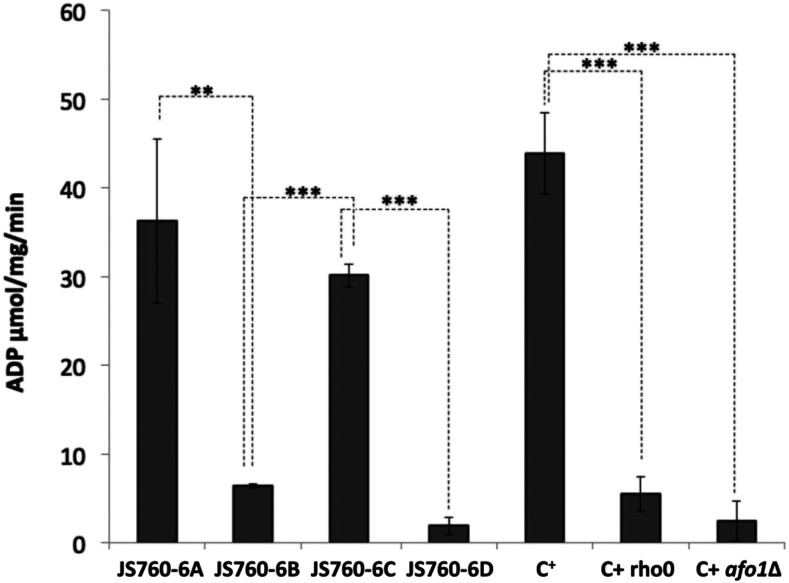
F_1_ ATPase activity measurements in strains of the tetrad JS760-6 and controls. All strains were grown in YPD to midlog phase, and submitochondrial particles were isolated and ATPase activity was measured as described by (Magri *et al.* 2010).

Of course, this is possible only as long as the mitochondrial adenine nucleotide transporter is intact - which is borne out by experimental results (Chen and Clark-Walker 2000). To test this hypothesis, we attempted to determine the activities of the soluble F_1_ ATPase in the strains of the tetrad JS760-6 and the C+, C+ *rho-zero*, and C+ *afo1*^-^ control strains. The method used to measure ATPase enzymatic activity was a coupled enzyme assay (see Materials and Methods) enabling the indirect quantitation of ADP using phosphoenol pyruvate as substrate and lactate dehydrogenase-mediated production of NAD^+^ as endpoint (Magri *et al.* 2010). Care was taken to avoid the influence of a possible ATP synthase contribution to the measurements (in the case of the respiratory-competent strains) by performing all measurements in the presence of antimycinA and oligomycin, which inhibits ATPsynthase but not the F_1_ ATPase reaction. As shown in Figure 7, F_1_ ATPase activity is high in the respiring strains, JS760-6A and JS760-6C, as well as in the control C+ strain, but significantly lower in the non-respiring strains JS760-6B, 6D, and the controls C+ *rho-zero* and C+ *afo1*^-^. The presence of the suppressor mutation does not increase F_1_ ATPase activity as shown in JS760-6D and the starting strain C+ *afo1*^-^. The conclusion is that the suppression of the slow growth phenotype and the restoration of the mitochondrial metabolic acitivity of *afo1*^-^ cells by the *ATP3**^G348T^* mutant allele is not due to an increase in ATPase activity. Therefore, a different (and at present unknown) mechanism underlies the suppressor activity of the *ATP3**^G348T^* allele. Nevertheless, the suppressor activity very probably requires assembly of an intact F_1_ ATPase structure as was discussed above, and at least minimal ATPase activity (Chen and Clark-Walker 2000; Lefebvre-Legendre *et al.* 2003).

**Figure 8:**
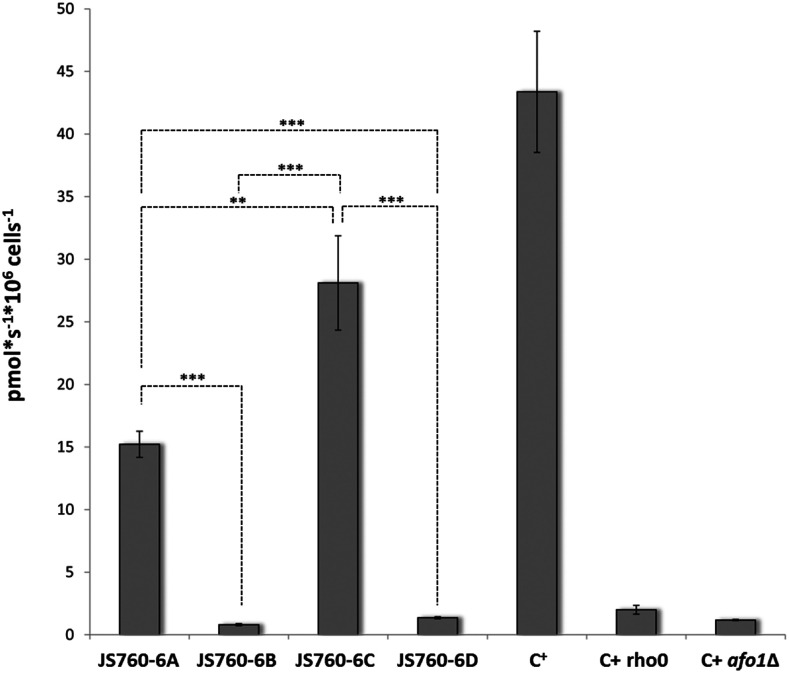
Oxygen uptake in the same strains as in Fig.7. All strains were grown in YPD to midlog phase, and oxygen consumption was measured immediately.

Another possible mechanism was an increase in oxygen uptake by the suppressed respiratory-deficient strain. Oxygen uptake was measured by high precision respirometry (Oroboros Oxygraph, see Materials and Methods). The result (Figure 8) clearly shows that the suppressor allele does not cause an increase in oxygen metabolism in the suppressed *afo1*^-^ respiratory deficient strain, which excludes the possibility that an increase in oxygen metabolism is the cause of the suppressor activtiy. The slightly lowered oxygen consumption of strain JS760-6A as compared to WT is presumably due to an intrinsic property of the suppressor allele *ATP3**^G348T^* but also due to the fact that the *ATP3**^G348T^* allele in a haploid cell leads to an increased frequency of loss of the mitochondrial genome. This means that possibly the cells used for the measurement were already a mixture of *rho-plus* and *rho-zero* cells. This is also indicated by the fact that the copy number of mitochondrial DNA is substantially lower in this strain than in the congenic WT strain (data not shown in detail).

### Spontaneous mutation frequencies in WT and C+ afo1^-^ strains

We next turned to the question of how it was possible that the suppressor mutations appeared so rapidly *de novo* in the *afo1*^-^ deleted strains. The generation of suppressor mutations (forming large colonies) was approximately equally frequent in the C+ strains discussed here and in the *afo1*^-^ deleted strains in the BY4741 background. Different suppressor mutations in the same gene (*ATP3*) with very similar genetic properties were found in diploid prototrophic rho-zero strains (Vowinckel, unpublished results).

Another hypothesis was that besides the strong selection for fast growing genetic suppressors, which occurs whenever the „slow“ strain (JS760-6B) is growing, an increased spontaneous mutation frequency could result in the formation of genetic suppressors in the *afo1*^-^deletion strain. Therefore, we measured mutation frequencies in the strains of the JS760-6 tetrad and in the WT and *rho-zero* controls. The purpose of these measurements was to clarify if the deletion of the *AFO1* gene or the *rho-zero* state of the strain can lead to a more rapid than WT occurrence of suppressor mutations by increasing the spontaneous mutation frequency.

**Figure 9:**
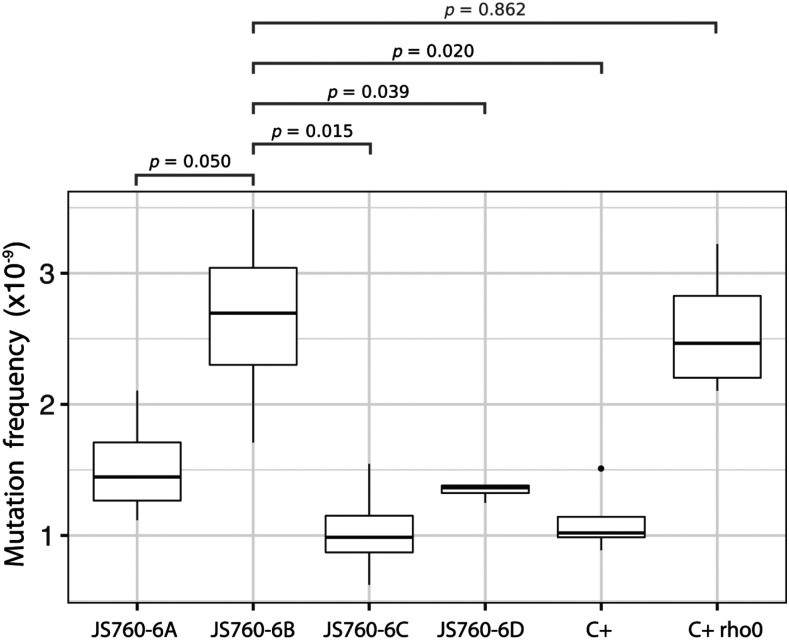
Spontaneous frequencies of point mutations (single nucleotide polymorphisms, SNPs) of the strains of tetrad JS760-6 and controls. Student’s p-values were used for pairwise comparisons of the mutation frequencies.

The results are shown in Figure 9. Genomic DNA was sequenced for the six strains shown in Figure 9 (ancestors) and 24 lines generated from the ancestors that were allowed to accumulate mutations. We found that the number of single nucleotide variants (SNVs) in the *afo1*^-^ deletion strain was twofold higher than in the WT strain (*P* < 0.05, *t*-test) but similar to the *rho-zero* control strain (*P* = 0.863, *t*-test). Note that the *afo1*^-^ deletion strain is also devoid of mitochondrial DNA as a consequence of the defect in mitochondrial protein synthesis. However, and most importantly, the JS760-6D strain (*afo1*^-^ and *ATP3**^G348^*^T^) which is also devoid of mitochondrial DNA, displays a spontaneous mutation frequency similar to WT. In order to confirm that all the mutations accumulated in a neutral fashion, we compared the numbers of SNVs occurring in the genic regions and the number of non-synonymous genic SNVs with the numbers expected (Liu and Zhang 2019; Sharp *et al.* 2018) in the absence of any selection during establishing the mutation accumulation lines. Those numbers were not significantly different: 71% *vs.* 74%; *P* > 0.10 Fisher’s exact test; and 73% *vs.* 76%; *P* > 0.10 Fisher’s exact test thus indicating the absence of selection in the SNV generation in the mutation accumulation lines.

The frequency of small indels was also higher in the *afo1*^-^-deleted strain compared to WT (*P* < 0.05, *t*-test) following a similar pattern as described for the SNVs.

We are presenting in detail only the SNVs here because all of the suppressor mutations found by us and by others were SNVs. Other aspects of this investigation of spontaneous mutation events including identity of the mutations found will be treated in a separate study.

The basic mutation frequency for point mutations (SNVs) in the unstressed haploid wild type strain C+ was about 1.5 × 10^−9^ mutations/(bp x replication round). This value confirms many textbook measurements (Alberts *et al.* 2008; Lodish 2016) but is nearly an order of magnitude higher than the one found with a different method in diploid yeast (Zhu *et al.* 2014). This may be explained by the fact observed earlier that single nucleotide mutations are less frequent in diploids than in haploids because of the additional possibilities for repair in diploids (Zhu *et al.* 2014).

As early as 1976, an increased reversion frequency in yeast *rho-zero* strains as compared with the congenic WT strains was observed (Flury *et al.* 1976). The strains were appropriately marked with reversible mutations and the revertant frequencies were determined. It was clear that some sort of mutation frequency increase was observed, however, this was not an unbiased, selection-free system.

(Lang and Murray 2008) determined forward mutation rates at the *CAN1* and *URA3* loci and estimated the per base mutation rates. These measurements were of course also not unbiased (unselected).

Taken together, the deletion of *AFO1* not only leads to loss of the mitochondrial genome but also to a significant increase in the spontaneous mutation frequency. An extragenic suppressor mutation generated in the *afo1*^-^ deletion strain restores the mutation frequency to levels observed for the wild type.

### Replicative lifespans

Finally, we wanted to check replicative lifespans in the newly made *afo1*^-^ and the suppressor mutations identified. Lifespans were determined by the microfluidics method (see Materials & Methods) in a tetrad of strains and controls in the BY4741 background and are presented in short form here. There was no significant change in the replicative lifespan due to *afo1*^-^ deletion mutation (data not shown). There seems to be a tendency to a short replicative lifespan in those members of the tetrad which carry the suppressor mutation. This result is at variance with our previous publication on the *afo1*^-^ mutant (Heeren *et al.* 2009).

There is presently no easy explanation, but likely (a) different suppressor mutation(s) must have been present in the deletion collection, although unknown at the time of the previous publication. Unexpected secondary mutations do occur relatively frequently in yeast deletion strains (Teng *et al*. 2013).

## Discussion

The results described here provide a tentative explanation for the occurrence of suppressor mutations in C+ *afo1*^-^ strains and suggest a mechanism that could lead to the observed phenotypes: rapid growth in the suppressed state, increase of the mutation frequency in the unsuppressed state and restoration of low mutation frequency (increased genomic stability) in the suppressed strain.

The unsuppressed *afo1*^-^ strain JS760-6B showed a twofold increase over WT in mutation frequency, but the suppressed strain JS760-6D showed a mutation frequency equal to WT (JS760-6C). The respiratory competent strain, JS760-6A, wich carries the *ATP3**^G348T^* allele, showed a mutation frequency similar to WT. The C^+^ rho-zero strain had a high mutation frequency equal to JS760-6B, but the starting strain, C+, showed a low mutation frequency that was comparable to the WT strain JS760-6C. We think it is possible that the large difference in mutation frequencies could contribute to the rapid occurrence of large colony variants after growing the *afo1*^-^ deleted strain on YPD or SD media. This tentative explanation is plausible, but cannot easily explain the apparent difference in reversion frequency (shown by the number of large colonies after re-streaking) between C+ *afo1*^-^ and C+ *rho-zero*, in spite of the fact that the mutation frequencies are similar (Figure 9).

An important question is the mechanism that leads to the increase in mutation frequency, and reversion to normal mutation frequency in the suppressed strain (JS760-6D). A possible explanation could be the following: The respiratory deficient strain JS760-6B just like the C+ rho-zero strain shows a partial defect in iron-sulfur cluster (ISC) synthesis leading to nuclear genome instability because both DNA synthesis and repair require ISC proteins (Dirick *et al.* 2014; Lill *et al.* 2014; Veatch *et al.* 2009). The authors noted increased specific growth rate in the suppressed strains (Dirick *et al.* 2014). However, they did not identify the genetic identity of the genes which harbor the suppressor alleles. Veatch *et al.* (Veatch *et al.* 2009) monitored the loss of heterozygosity in diploid yeast of the BY4743 background. In the present communication, forward formation of SNVs is measured in non-coding as well as coding parts of the genome and in positions where the mutations created are synonymous as well as non-synonymous. Comparing these results, we conclude that the mutations measured originated in the absence of selection. The mutational events monitored here (SNVs) are of the kind that were found to lead to the suppressor mutations found in respiratory deficient *S. cerevisiae* and *K. lactis* investigations not only in the present communication, but also in (Chen and Clark-Walker 1999, 1995, 2000). Loss of heterozygosity, which was also found in respiratory deficient diploid yeast strains (Veatch *et al.* 2009) or large chromosomal rearrangements are less likely to create dominant suppressors of the slow growth phenotype of respiratory-deficient yeast.

Taken together, the results presented here contribute to understanding the physiology of yeast respiratory deficient mutants. The phenotypes observed depend not on a defect in ATP production, but on a different mitochondrial defect, possibly in ISC protein synthesis, which would be in line with to the observed genetic instability. However, an intact F_1_ ATPase complex is apparently needed (this is also clear from the work of Clark-Walkeret al., (Chen and Clark-Walker 2000)), even if the actual ATPase activtiy is low (Figure 7). So, perhaps the intact soluble ATPase complex could have a second function independent of splitting of ATP.

The new insights presented here could help to understand mitochondrial physiology in cells with respiratory deficiencies.
